# Multiple pathways contribute to the pathogenesis of Huntington disease

**DOI:** 10.1186/1750-1326-1-19

**Published:** 2006-12-16

**Authors:** Shihua Li, Xiao-Jiang Li

**Affiliations:** 1 Department of Human Genetics, Emory University School of Medicine, 615 Michael Street, Atlanta, GA 30322, USA

## Abstract

Huntington disease (HD) is caused by expansion of a polyglutamine (polyQ) domain in the protein known as huntingtin (htt), and the disease is characterized by selective neurodegeneration. Expansion of the polyQ domain is not exclusive to HD, but occurs in eight other inherited neurodegenerative disorders that show distinct neuropathology. Yet in spite of the clear genetic defects and associated neurodegeneration seen with all the polyQ diseases, their pathogenesis remains elusive. The present review focuses on HD, outlining the effects of mutant htt in the nucleus and neuronal processes as well as the role of cell-cell interactions in HD pathology. The widespread expression and localization of mutant htt and its interactions with a variety of proteins suggest that mutant htt engages multiple pathogenic pathways. Understanding these pathways will help us to elucidate the pathogenesis of HD and to target therapies effectively.

## Background

Huntington's disease (HD) was first described by the American physician George Huntington in 1872. The disease is a genetic disorder of the central nervous system, with symptoms usually consisting of uncontrolled movements, emotional disturbances, and mental deterioration [1,2]. Psychiatric abnormalities, including depression, anxiety, apathy, and irritability, are often an early manifestation of HD, appearing before the characteristic neurologic symptoms of overt chorea, or spasmodic movements of the limbs and facial muscles [3,4]. Patients also demonstrate cognitive deficits or dementia as well as weight loss. The age at onset is variable, but most HD patients first show symptoms between the ages of 30 and 40 years [5,6]. Clinical features develop progressively, with an increase in choreic movements, dementia, and other motor deficits including dystonia and rigidity. HD terminates in death within 10–20 years after initial symptoms appear. While there are medications to help manage the signs and symptoms of HD, treatments that can actually prevent the physical and mental decline associated with HD are lacking.

HD is inherited as an autosomal dominant condition and is found in every country of the world. In the United States alone, about 30,000 people have HD, making its prevalence about 1 in every 10,000 persons. At least 150,000 others carry a 50 percent risk of developing the disease, and thousands more of their relatives live with the possibility that they might develop HD.

The hunt for the HD gene involved an intense molecular genetics research effort by investigators cooperating around the globe. For 10 years, scientists were focused on a segment of chromosome 4p16.3, and they succeeded in isolating the HD gene in 1993 [7]. The genetic defect responsible for the disease is expansion of a CAG repeat in the gene coding for the HD protein, huntingtin (htt). This CAG repeat is an unstable triplet repeat DNA sequence, and its length is inversely correlated with the age at onset of disease, especially in juvenile HD cases, when the repeat length is often >60 CAG units [8-10]. Expanded CAG repeats have been found in 8 other inherited neurodegenerative diseases, as well, including spinocerebellar ataxia (SCA) and spinobulbar muscular atrophy (SBMA) [11-13]. It is now clear that expansion of this repeat in various genes can cause distinct neurodegenerative pathology in different disorders.

The CAG repeat is translated into a polyglutamine (polyQ) domain in the disease proteins. Human htt is a large protein consisting of 3144 amino acids. A normal polyQ domain, which in htt begins at amino acid position 18, typically contains 11–34 glutamine residues in unaffected individuals, but this expands to more than 37 glutamines in HD patients. The length of the polyQ repeat varies among species. For example, mouse htt has 7 glutamines, whereas pufferfish htt contains only 4 [14], which suggests that the polyQ domain may not be essential, but that it can regulate protein function. Consistently, deletion of the CAG repeat in the HD gene only results in subtle behavioral and motor phenotypes in mice [15].

Htt is ubiquitously expressed in the brain and body and distributed in various subcellular regions [16-19]. Its sequences do not show homology to other proteins of known function. One structural feature of htt that has been identified is the presence of HEAT repeats [20], which are sequences of ~40 amino acids that occur multiple times within a given protein and are found in a variety of proteins involved in intracellular transport and chromosomal segregation [21]. Several lines of evidence also suggest that htt is involved in intracellular trafficking and various cellular functions. For example, htt is associated with a number of subcellular organelles [16-18,22]. Consistent with this, htt is known to interact with a variety of proteins that can be grouped according to whether they are involved in gene transcription, intracellular signaling, trafficking, endocytosis, or metabolism [14,19]. Identification of these htt-interacting proteins suggests that htt may function as a scaffold involved in coordinating sets of proteins for signaling processes and intracellular transport.

The essential role of htt has been established using HD gene knockout mice. In this model, the absence of htt causes cell degeneration and embryonic lethality [23-25]. Conditional knockout mice also show degeneration in adult cells [26]. These observations have led to the theory that a loss of htt function may contribute to the neuropathology of HD [27]. However, there is more evidence to support the theory wherein mutant htt gains a toxic function. For example, heterozygous HD knockout mice are known to live normally. Further, identification of the HD gene has allowed for generation of various animal models in which mutant htt is expressed in the presence of endogenous normal htt, and these transgenic mice still develop neurological symptoms and die early, even when endogenous normal htt is expressed at the normal levels [28,29]. In addition, mutant htt can rescue the embryonic lethal phenotype of htt-null mice [30], which also suggests the HD mutation can lead to neuronal toxicity, independent of the essential function of htt.

### Neuropathology of Huntington's disease

Despite its widespread distribution, mutant htt causes selective neurodegeneration, which occurs preferentially and most prominently in the striatum and deeper layers of the cortex in the early stages of HD [31]. In advanced stages, other brain regions, such as the hippocampus, hypothalamus, cerebellum, amygdala, and some thalamic nuclei, are also affected. Among these other brain regions, the lateral tuberal nucleus of the hypothalamus exhibits severe atrophy [32].

The neurons that are most severely affected in HD are striatal projection neurons, which send their axons to different brain regions. These are the GABAergic medium-sized spiny neurons (MSNs), and they constitute 95% of all striatal neurons. MSNs receive abundant glutamatergic input from the cortex and primarily innervate the substantia nigra and globus pallidus. Thus, their preferential loss in HD is thought to be the result of glutamate excitotoxicity. Consistently, there is a relative sparing of interneurons that colocalize somatostatin, neuropeptide Y, and NADPH diaphorase, as well as of cholinergic interneurons and a subclass of GABAergic neurons that contain parvalbumin [31,33,34].

Another important pathological feature in the postmortem brains of HD patients is gliosis [35-37]. Reactive glia or gliosis often occurs in response to neuronal injury. For example, neuronal degeneration is evidenced by a dramatic elevation in the density of large glia [38]. Marked astrogliosis and microgliosis were observed in caudate and internal capsule samples of HD patients, but not in normal brain. In the striatum and cortex, reactive microglia also occurred in all grades of pathology, accumulated with increasing grade, and grew in density in relation to the degree of neuronal loss [35,37]. Thus, reactive microglia were considered to be an early response to changes in neuropil [37]. While reactive gliosis does represent an early neuropathological event in HD, glial pathology can also impact neuronal viability. Indeed, gliosis is a pathological feature in several HD mouse models that lack overt neuronal cell degeneration. These models include transgenic mice expressing N-terminal mutant htt [39-41] and knock-in mice that express full-length mutant htt [42,43].

Since the discovery of the HD gene, various antibodies to htt have been generated to characterize the distribution of mutant htt. Immunostaining of brains from transgenic mice that express mutant htt revealed nuclear inclusions [28]. Similar nuclear inclusions were then identified in the brains of HD patients [44,45]. Subsequently, the accumulation of expanded polyQ-containing proteins in the nucleus and nuclear inclusions were found to be common pathological features of other polyglutamine diseases [11-13]. The role of these nuclear inclusions in HD remains controversial, since their formation is correlated with disease progression, but is not associated with neuronal degeneration [45-47]. Further, several studies have shown that htt inclusions are protective against htt toxicity in cultured cells [48,49]. Despite the controversy surrounding their exact roles, htt inclusions reflect protein misfolding caused by expanded polyQ domains and represent a pathological hallmark for the accumulation of toxic mutant htt. It is also noteworthy that normal htt is predominantly localized in the cytoplasm, whereas mutant htt with its expanded polyQ domain accumulates in the nucleus. Therefore, nuclear inclusions reflect the aberrant accumulation of mutant htt in the nucleus, as well. Importantly, HD also features abundant cytoplasmic aggregates localized in the neuronal processes (neuropil aggregates), including axons and dendrites [29,45,50-54]. In the early stage of disease, the brains of HD patients contain more dystrophic neurites or neuropil aggregates than nuclear inclusions [44,45]. In addition, the progressive formation of neuropil aggregates is correlated with disease progression in transgenic mice [50-52,54], and neuropil aggregates are associated with axonal degeneration in HD mouse brains [40,52]. Taken together, the localization of htt aggregates in the nucleus and neuronal processes reveals that mutant htt elicits toxicity in both the nucleus and cytoplasm.

A number of mouse models have provided in vivo evidence for the pathology of HD. Several transgenic HD mice were generated using either the human htt promoter or neuronal promoters. For example, transgenic mice R6/2 express exon1 htt with 115–150 glutamine repeats (115–150Q) under the control of the human HD gene promoter [28]. YAC transgenic mice use the human HD gene promoter to drive the expression of full-length mutant htt [55,56]. N171-82Q transgenic mice express the first 171 amino acids with 82Q under the neuronal prion promoter [29]. These transgenic mice have been widely studied and found to have neurological and behavioral phenotypes similar to those of HD patients. There are also HD repeat knock-in mouse models, which are generated by inserting an expanded repeat into the endogenous mouse HD gene [43,57-59]. However, most HD mouse models do not show the overt neurodegeneration seen in human HD patients, even though some models display severe neurological symptoms and early death [28,29]. It is possible that the short life span of the mouse does not allow sufficient time for the development of obvious neurodegeneration, although some earlier pathological events do occur.

HD mouse models also suggest that small htt fragments containing expanded polyQ are more toxic than larger fragments. This fits with the finding that small N-terminal htt fragments are misfolded and form aggregates and inclusions in the brains of HD patients [44,45]. It is evident that proteolysis of htt generates multiple N-terminal htt fragments in HD repeat knock-in mouse brain [60]. A number of protease cleavage sites, including those for capsase-3, cspase-6, calpain, and unknown aspartic protease, have been found within the first 550 amino acids of htt [56,61-64]. However, most studies used transfected proteins to identify these cleavage sites, and the nature of toxic N-terminal htt fragments generated organically in the HD brain is still being explored. It is likely that the proteolysis of full-length htt generates a number of N-terminal htt fragments. The decreased activities of the proteasomes and chaperones, which are responsible for clearing out misfolded and toxic peptides, promote the accumulation of htt fragments in aged neurons. In the meantime, an expanded polyglutamine tract causes them to misfold and aggregate in the nucleus and neuronal processes. The accumulation of mutant htt in the nucleus and neuronal processes therefore suggests that these subcellular regions are the primary sites for mutant htt to elicit its toxicity.

### Nuclear effect of mutant huntingtin

The nuclear inclusions of mutant htt led investigators to study the mechanisms for this phenomenon. Although some immunostaining and nuclear fractionation studies have shown that normal htt is also localized in the nucleus [65,66], it is clear that the majority remains in the cytoplasm. Moreover, nuclear htt aggregates can only be recognized by antibodies against the N-terminal region of htt [44,45]. Furthermore, isolation of nuclear fractions from HD knock-in mice, which express full-length mutant htt under the endogenous mouse HD gene, provides evidence that multiple N-terminal htt fragments accumulate in the nucleus [60]. The association between nuclear accumulation of mutant htt and disease progression is clear from several HD mouse models. In HD knock-in mouse models, mutant htt accumulates preferentially in the nuclei of striatal neurons and forms more prominent aggregates as the disease progresses [43,58]. A progressive phenotype is also associated with the nuclear accumulation of an amino-terminal cleavage fragment in a transgenic mouse model with inducible expression of full-length mutant huntingtin [67]. Targeting mutant htt with nuclear localization sequences to direct mutant htt in the nucleus of mouse brains produces neurological phenotypes [68,69]. Furthermore, prevention of htt cleavage by mutating the caspase-6 site can alleviate neurological phenotypes and delay the nuclear accumulation of mutant htt in YAC transgenic mice [56].

Studies of N-terminal htt fragments have failed to find that these fragments contain nuclear localization sequences. Thus, N-terminal htt fragments may passively enter the nucleus, but expanded polyQ repeats prevent their export from the nucleus [70]. The presence of mutant htt fragments in the nucleus and various cleavage sites in the N-terminal region of htt [61-64,71] also support the notion that proteolysis of htt leads to generation of toxic htt fragments. Consistently, smaller N-terminal htt fragments appear to be more toxic than large-sized fragments in both cultured cells [72] and transgenic animals [28,29,40]. For example, R6/2 mice that express exon1 (1–67 amino acids) with 115–150Q show more severe neurological phenotypes than transgenic mice that express longer-fragment (N171-82Q) or full-length mutant htt [28,29,56]. In addition, transgenic mice expressing smaller htt fragments show more abundant htt inclusions in the nucleus than those expressing longer htt fragments.

The aberrant nuclear accumulation of mutant htt is likely to cause gene transcriptional dysregulation. Indeed, several nuclear transcription factors are found to bind htt [19,73]. Of these, the coactivators cAMP response element-binding protein(CREB)-binding protein (CBP) and specificity protein 1 (Sp1) are particularly important for neuronal function. Deletion of CREB in the brain causes selective neurodegeneration in the hippocampus and striatum [74]. Many neuronal genes that lack a TATA box require Sp1 for their transcription [75]. Dysregulation of gene expression mediated by CBP and Sp1 have been found in HD mouse brains [76].

The interactions of mutant htt with transcription factors may occur at various binding sites. Many transcription factors contain a polyQ-rich domain. Since CBP is recruited into aggregates formed by different polyQ proteins, such as the androgen receptor [77], the SCA3 [78], and the DRPLA [79] proteins, it has been thought that the polyQ domain is the binding site to interact with other polyQ proteins. In support of this idea, a number of transcription factors containing polyQ or proline-rich domains, including CBP [79,80], TBP [81,82], and TAF130 [83], have been found in nuclear polyQ inclusions. However, subsequent studies showed that the acetyltransferase domain in CBP interacts with htt [78,80], which led to the finding that inhibition of histone deacetylase (HDAC) or promotion of histone acetylation ameliorates neurodegeneration in cellular and fly models [80] and motor deficits in a mouse model of HD [84]. The colocalization of some transcription factors in nuclear polyQ inclusions also led to the idea that recruitment of transcription factors into polyQ inclusions reduces the level of these transcription factors. After examining several HD mouse models, however, researchers were unable to find decreased levels of CBP in symptomatic mouse brains [54,85]. In addition, altered expression of a number of genes was not necessarily associated with the formation of htt aggregates in HD mice [76] and could occur in cell models in the absence of nuclear inclusions [86,87]. Thus, it is likely that soluble or misfolded htt may interact with transcription factors to alter transcriptional activity. This idea is further supported by the finding that soluble mutant htt reduces the binding of Sp1 to DNA [88,89].

Several other important transcription factors are also implicated for their interactions with htt in the nucleus. TAF130, which is an important transcription factor that binds TBP and is involved in Sp1 and CREB-dependent gene transcription, binds htt [88] and other polyQ proteins, such as the DRPLA and SCA3 proteins [83]. TBP, which is a basal transcriptional factor containing a polyQ stretch, is also found to colocalize with polyQ inclusions [81,82] and to associate with htt in vitro [90]. In addition, htt interacts with p53 in the nucleus to affect cell viability [91]. Recent studies also show that mutant htt can affect PGC-1alpha (peroxisome proliferator-activated receptor gamma coactivator 1alpha), which transduces many physiological stimuli into specific metabolic programs by stimulating mitochondrial activity. Lack of this transcription factor causes degeneration in different types of cells, including striatal neurons [92,93]. Cui et al's study showed that mutant htt associates with the promoter of the PGC-1alpha gene to affect its expression, thereby mediating striatal degeneration [94]. The interference of PGC-1alpha function is also observed in brown fat tissues in HD transgenic mice [95]. These studies suggest PGC-1alpha as a new therapeutic target in HD. Combined, there is ample evidence that mutant htt acts in the nucleus to affect gene transcription.

### Cytoplasmic effect of mutant huntingtin

Earlier studies have reported that mutant htt increases caspase activity [96-99] and affects various signaling pathways [100-104]. These findings suggest that mutant htt also acts in the cytoplasm to affect cellular functions. Thus began an intensive search to reveal the interactions between htt and cytoplasmic proteins. By means of the yeast two-hybrid screen and in vitro binding assays, a number of cytoplasmic proteins were found to interact with htt [14,18,105]. Of these, htt-associated protein 1 (HAP1) and htt-interacting protein 1 (HIP1) have been studied extensively. Both proteins may be involved in intracellular trafficking. HAP1 binds more tightly to mutant htt than to normal htt [106,107]. HAP1 also associates with both dynactin p150, which is involved in microtubule-dependent retrograde transport [108,109], and kinesin light chain 2 [110], which is involved in anterograde transport. Several studies suggest that HAP1 participates in the trafficking or endocytosis of membrane receptors, including those for epidermal growth factor [111], type 1 inositol (1,4,5)-triphosphate receptor (InsP3R1) [103], GABA [112], and nerve growth factor (NGF) [113]. Like htt, HAP1 is located at various subcellular sites, including microtubules and synaptic vesicles in axonal terminals [22]. Mice lacking HAP1 often die at postnatal day 3 [114,115], which is likely as a result of neuronal degeneration in the hypothalamus [115]. The hypothalamic function of HAP1 appears to be critical for feeding behavior and metabolism [116], and its dysfunction may contribute to hypothalamic pathology or degeneration in HD [32,115,117].

HIP1 is also important for assembly and function of the cytoskeleton and endocytosis [118] and binds clathrin and alpha-adaptin subunit AP-2 [119-121]. The interactions of HIP1 with these proteins may constitute a protein complex involved in clathrin-mediated endocytosis. Unlike HAP1, HIP1 binds mutant htt weakly [118]. This suggests that HIP1 requires interaction with htt for normal function, whereas dissociation from mutant htt may impair its function.

Although the interactions of htt with HAP1, HIP1, and other cytoplasmic proteins suggest that htt is involved in intracellular trafficking, more compelling evidence has come from the studies of trafficking function in cells that express mutant htt. Recent studies show that normal *Drosophila *htt functions in the axonal transport pathway and that polyQ expansion causes soluble htt to recruit more microtubule transporter proteins, thereby reducing the soluble pool of these proteins in axons [122]. In cultured neurons, htt is involved in HAP1-associated axonal transport of brain-derived neurotrophic factor (BDNF) both anterogradely and retrogradely, and mutant htt disrupts this transport [107]. Trushina et al (2004) also found that expression of full-length mutant htt impaired vesicular and mitochondrial trafficking in mouse neurons [123]. Similarly, expanded polyQ in the first exon of htt can cause axonal abnormalities prior to cell body degeneration in *Caenorhabditis elegans*, even in the absence of cell body aggregates [124]. Also, expressing polyQ proteins in cultured neurons shows that mutant htt can affect axonal transport [125].

Because of the limited space of neuronal processes, neuropil aggregates themselves may physically impair trafficking or affect neurotransmitter release [126,127]. Recently, htt aggregates were found to affect the trafficking of mitochondria [128], suggesting that neuropil aggregates could also impair the trafficking of other organelles. There is strong evidence for the pathological role of polyQ aggregates in axonal degeneration. As mentioned previously, abundant dystrophic neurites are evident in presymptomatic postmortem HD patient brains in the cortex and the striatum, the two areas most affected in HD [44,45]. Dystrophic neurites are abnormal structures outside the cell body and are potentially derived from degenerated axons or dendrites. In HD repeat knock-in mice, which do not show obvious neurological phenotypes, large htt aggregates are found in degenerating axons and axonal terminals [40,52].

The finding of axonal dysfunction or degeneration in various HD models provides a compelling argument that axonal dysfunction is an early neuropathological event in HD [52,122,125,129]. Degeneration of axons often precedes the death of the cell body and is commonly associated with a variety of neurodegenerative disorders, including Wallerian degeneration, Alzheimer's disease, and Parkinson's disease [130,131]. The degeneration of the distal ends of axons can lead to defective neuronal interaction, abnormal synaptic transmission, and an impaired supply of growth factors to the cell body, eventually causing the loss of the neuronal body.

### Cell-cell interactions and Huntington's disease

Cell-cell interactions constitute the complex circuitry that regulates the normal function of neurons in various brain regions. Previous studies have focused on the autonomic effect of mutant htt on neuronal function, while scant attention has been paid to the role of cell-cell interactions in HD pathogenesis. Gu et al created conditional HD mice that express exon1 mutant htt in discrete neuronal populations. They found that mutant htt forms aggregates in a cell-autonomous manner. However, progressive motor deficits and cortical neuropathology are observed only when mutant htt is expressed in multiple neuronal types, not when mutant htt is restricted to cortical pyramidal neurons [41]. Since the transgenic htt is expressed under a neuronal promoter in their study, their findings provide compelling evidence for involvement of neuronal cell interactions in HD pathology.

Glial cells constitute 90% of the cells in the brain and provide nutrition, growth factors, and structural support for neurons to survive and function normally. They also protect against excitotoxicity by removing excess excitatory neurotransmitters from the extracellular space [132-134]. This protective function may be particularly relevant to the neuropathology of HD, since excitotoxicity has been a long-standing theory to account for the pathogenesis of HD [135-137]. As discussed above, MSNs in the striatum are largely innervated by glutamatergic axons and are preferentially degenerated in HD. Glutamate activates ionotropic glutamate receptors, specifically the N-methyl-D-aspartate (NMDA) and non-NMDA receptors (ie, AMPA/kainate). Overstimulation of glutamate receptors by high levels of extracellular glutamate induces excitotoxicity [138]. Administration of NMDA receptor agonists to the striatum of animals causes a selective loss of MSNs and produces neurological symptoms similar to those seen in HD patients [136,139], whereas NMDA receptor antagonists effectively reduce excitotoxicity in HD animal models [140]. Furthermore, HD transgenic mouse models show increased NMDA receptor activity in neurons [102,141,142]. Because of the abundant glutamatergic innervation to MSNs, cell-cell interactions may be particularly important for the vulnerability of MSNs to extracellular glutamate.

Clearance of extracellular excitatory neurotransmitters is largely carried out by astrocytes, which are the major subtype of glia. These cells contain membrane receptors (GLT-1 and GLAST) that transport extracellular glutamate into the cytoplasm, where glutamate is subsequently metabolized by glutamine synthase [143]. Some studies have suggested that the function of GLT-1 is impaired in HD. HD mouse brains have an increased extracellular glutamate concentration and a reduced expression level of GLT-1 [144,145]. Transgenic mutant htt in *Drosophila *glia reduces the expression of glutamate transporter and shortens the life span of the fly [146]. Shin et al provided evidence that mutant htt is also expressed in glial cells in the brains of both HD mice and HD patients. They further demonstrated that mutant htt reduces glial glutamate uptake, as well as the protection it confers against htt-mediated neurotoxicity [147]. These studies suggest that glia-neuron interactions also play important roles in the pathogenesis of HD.

### Summary

Since the discovery of the gene mutation in HD, there have been great strides towards elucidating the pathogenesis of this disease. It has become clear that polyQ expansion can cause mutant htt to misfold and to aggregate. Misfolded htt abnormally interacts with a variety of proteins and also accumulates in the nucleus and neuronal processes. Furthermore, it is evident that proteolysis of htt is required for the aggregation and misfolding of mutant htt. Accordingly, many cleavage sites have been found in the N-terminal region of htt, and tremendous efforts have been put forth to find a means of blocking the generation of toxic htt fragments. The present review focuses on the effects of mutant htt in the nucleus and cytoplasm. Given its localization in both the nucleus and neuronal processes and its interactions with a variety of proteins, mutant htt is likely to affect a number of targets. Thus, it is conceivable that mutant htt engages multiple pathogenic pathways.

An important issue facing researchers is how to sort out the major pathogenic pathways as targets for developing therapeutic strategies. For example, which is the more critical for neuronal dysfunction and neurodegeneration, the nuclear or the cytoplasmic effect of mutant htt? Answering this question would require a better understanding than we currently possess of mutant htt's effects in different types of cells and at different stages of the disease. The preferential localization of mutant htt in the nucleus of striatal neurons suggests that the nuclear effect of mutant htt or gene transcriptional dysregulation may affect neuronal function at the early stage of the disease. In other types of cells, mutant htt in the cytoplasm could also impair neuronal function without showing nuclear accumulation. In the case of mitochondrial dysfunction and excitotoxicity, which represent an early theory of HD pathogenesis, mutant htt may act in the nucleus to affect the expression of mitochondrial proteins. It can also directly impair mitochondrial function in the cytoplasm. In addition, cell-cell interactions and circuitry are critical for the selective toxicity of mutant htt. For example, the function of medium spiny neurons in the striatum is regulated to a great extent by BDNF and glutamate input from cortical neurons. Their vulnerability is also influenced by the ability of glial cells to protect against excitotoxicity as well as the effect of mutant htt on NMDA receptors. It is therefore likely that mutant htt affects multiple targets at different levels, leading to the selective neurodegeneration of HD. Understanding how mutant htt mediates these pathological pathways would help us to find effective treatments for the disease.

## Acknowledgements

The work from the authors' laboratory was supported by grants from the NIH (AG19206, NS045016, NS36232, NS41669). Due to space limitations, we apologize to those investigators whose work could not be included in this review.

## References

[B1] Martin JB, Gusella JF (1986). Huntington's disease. Pathogenesis and management. N Engl J Med.

[B2] Harper PS, Saunders WB (1991). Huntington's disease.

[B3] Folstein SE, Leigh RJ, Parhad IM, Folstein MF (1986). The diagnosis of Huntington's disease. Neurology.

[B4] Shiwach RS, Norbury CG (1994). A controlled psychiatric study of individuals at risk for Huntington's disease. Br J Psychiatry.

[B5] Chandler JH, Reed TE, Dejong RN (1960). Huntington's chorea in Michigan. III. Clinical observations. Neurology.

[B6] Foroud T, Gray J, Ivashina J, Conneally PM (1999). Differences in duration of Huntington's disease based on age at onset. J Neurol Neurosurg Psychiatry.

[B7] HD collaborative research group (1993). A novel gene containing a trinucleotide repeat that is expanded and unstable on Huntington's disease chromosomes. The Huntington's Disease Collaborative Research Group. Cell.

[B8] Andrew SE, Goldberg YP, Kremer B, Telenius H, Theilmann J, Adam S, Starr E, Squitieri F, Lin B, Kalchman MA (1993). The relationship between trinucleotide (CAG) repeat length and clinical features of Huntington's disease. Nat Genet.

[B9] Duyao M, Ambrose C, Myers R, Novelletto A, Persichetti F, Frontali M, Folstein S, Ross C, Franz M, Abbott M (1993). Trinucleotide repeat length instability and age of onset in Huntington's disease. Nat Genet.

[B10] Snell RG, MacMillan JC, Cheadle JP, Fenton I, Lazarou LP, Davies P, MacDonald ME, Gusella JF, Harper PS, Shaw DJ (1993). Relationship between trinucleotide repeat expansion and phenotypic variation in Huntington's disease. Nat Genet.

[B11] Zoghbi HY, Orr HT (2000). Glutamine repeats and neurodegeneration. Annu Rev Neurosci.

[B12] Gatchel JR, Zoghbi HY (2005). Diseases of unstable repeat expansion: mechanisms and common principles. Nat Rev Genet.

[B13] Butler R, Bates GP (2006). Histone deacetylase inhibitors as therapeutics for polyglutamine disorders. Nat Rev Neurosci.

[B14] Harjes P, Wanker EE (2003). The hunt for huntingtin function: interaction partners tell many different stories. Trends Biochem Sci.

[B15] Clabough EB, Zeitlin SO (2006). Deletion of the triplet repeat encoding polyglutamine within the mouse Huntington's disease gene results in subtle behavioral/motor phenotypes in vivo and elevated levels of ATP with cellular senescence in vitro. Hum Mol Genet.

[B16] Gutekunst CA, Levey AI, Heilman CJ, Whaley WL, Yi H, Nash NR, Rees HD, Madden JJ, Hersch SM (1995). Identification and localization of huntingtin in brain and human lymphoblastoid cell lines with anti-fusion protein antibodies. Proc Natl Acad Sci U S A.

[B17] DiFiglia M, Sapp E, Chase K, Schwarz C, Meloni A, Young C, Martin E, Vonsattel JP, Carraway R, Reeves SA (1995). Huntingtin is a cytoplasmic protein associated with vesicles in human and rat brain neurons. Neuron.

[B18] Sharp AH, Loev SJ, Schilling G, Li SH, Li XJ, Bao J, Wagster MV, Kotzuk JA, Steiner JP, Lo A (1995). Widespread expression of Huntington's disease gene (IT15) protein product. Neuron.

[B19] Li SH, Li XJ (2004). Huntingtin-protein interactions and the pathogenesis of Huntington's disease. Trends Genet.

[B20] Takano H, Gusella JF (2002). The predominantly HEAT-like motif structure of huntingtin and its association and coincident nuclear entry with dorsal, an NF-kB/Rel/dorsal family transcription factor. BMC Neurosci.

[B21] Neuwald AF, Hirano T (2000). HEAT repeats associated with condensins, cohesins, and other complexes involved in chromosome-related functions. Genome Res.

[B22] Gutekunst CA, Li SH, Yi H, Ferrante RJ, Li XJ, Hersch SM (1998). The cellular and subcellular localization of huntingtin-associated protein 1 (HAP1): comparison with huntingtin in rat and human. J Neurosci.

[B23] Duyao MP, Auerbach AB, Ryan A, Persichetti F, Barnes GT, McNeil SM, Ge P, Vonsattel JP, Gusella JF, Joyner AL (1995). Inactivation of the mouse Huntington's disease gene homolog Hdh. Science.

[B24] Nasir J, Floresco SB, O'Kusky JR, Diewert VM, Richman JM, Zeisler J, Borowski A, Marth JD, Phillips AG, Hayden MR (1995). Targeted disruption of the Huntington's disease gene results in embryonic lethality and behavioral and morphological changes in heterozygotes. Cell.

[B25] Zeitlin S, Liu JP, Chapman DL, Papaioannou VE, Efstratiadis A (1995). Increased apoptosis and early embryonic lethality in mice nullizygous for the Huntington's disease gene homologue. Nat Genet.

[B26] Dragatsis I, Levine MS, Zeitlin S (2000). Inactivation of Hdh in the brain and testis results in progressive neurodegeneration and sterility in mice. Nat Genet.

[B27] Cattaneo E, Rigamonti D, Goffredo D, Zuccato C, Squitieri F, Sipione S (2001). Loss of normal huntingtin function: new developments in Huntington's disease research. Trends Neurosci.

[B28] Davies SW, Turmaine M, Cozens BA, DiFiglia M, Sharp AH, Ross CA, Scherzinger E, Wanker EE, Mangiarini L, Bates GP (1997). Formation of neuronal intranuclear inclusions underlies the neurological dysfunction in mice transgenic for the HD mutation. Cell.

[B29] Schilling G, Becher MW, Sharp AH, Jinnah HA, Duan K, Kotzuk JA, Slunt HH, Ratovitski T, Cooper JK, Jenkins NA, Copeland NG, Price DL, Ross CA, Borchelt DR (1999). Intranuclear inclusions and neuritic aggregates in transgenic mice expressing a mutant N-terminal fragment of huntingtin. Hum Mol Genet.

[B30] Hodgson JG, Smith DJ, McCutcheon K, Koide HB, Nishiyama K, Dinulos MB, Stevens ME, Bissada N, Nasir J, Kanazawa I, Disteche CM, Rubin EM, Hayden MR (1996). Human huntingtin derived from YAC transgenes compensates for loss of murine huntingtin by rescue of the embryonic lethal phenotype. Hum Mol Genet.

[B31] Vonsattel JP, Myers RH, Stevens TJ, Ferrante RJ, Bird ED, Richardson EP (1985). Neuropathological classification of Huntington's disease. J Neuropathol Exp Neurol.

[B32] Kremer HP, Roos RA, Dingjan G, Marani E, Bots GT (1990). Atrophy of the hypothalamic lateral tuberal nucleus in Huntington's disease. J Neuropathol Exp Neurol.

[B33] Ferrante RJ, Kowall NW, Beal MF, Richardson EP, Bird ED, Martin JB (1985). Selective sparing of a class of striatal neurons in Huntington's disease. Science.

[B34] Graveland GA, Williams RS, DiFiglia M (1985). Evidence for degenerative and regenerative changes in neostriatal spiny neurons in Huntington's disease. Science.

[B35] Myers RH, Vonsattel JP, Paskevich PA, Kiely DK, Stevens TJ, Cupples LA, Richardson EP, Bird ED (1991). Decreased neuronal and increased oligodendroglial densities in Huntington's disease caudate nucleus. J Neuropathol Exp Neurol.

[B36] Singhrao SK, Thomas P, Wood JD, MacMillan JC, Neal JW, Harper PS, Jones AL (1998). Huntingtin protein colocalizes with lesions of neurodegenerative diseases: An investigation in Huntington's, Alzheimer's, and Pick's diseases. Exp Neurol.

[B37] Sapp E, Kegel KB, Aronin N, Hashikawa T, Uchiyama Y, Tohyama K, Bhide PG, Vonsattel JP, DiFiglia M (2001). Early and progressive accumulation of reactive microglia in the Huntington disease brain. J Neuropathol Exp Neurol.

[B38] Rajkowska G, Selemon LD, Goldman-Rakic PS (1998). Neuronal and glial somal size in the prefrontal cortex: a postmortem morphometric study of schizophrenia and Huntington disease. Arch Gen Psychiatry.

[B39] Ishiguro H, Yamada K, Sawada H, Nishii K, Ichino N, Sawada M, Kurosawa Y, Matsushita N, Kobayashi K, Goto J, Hashida H, Masuda N, Kanazawa I, Nagatsu T (2001). Age-dependent and tissue-specific CAG repeat instability occurs in mouse knock-in for a mutant Huntington's disease gene. J Neurosci Res.

[B40] Yu ZX, Li SH, Evans J, Pillarisetti A, Li H, Li XJ (2003). Mutant huntingtin causes context-dependent neurodegeneration in mice with Huntington's disease. J Neurosci.

[B41] Gu X, Li C, Wei W, Lo V, Gong S, Li SH, Iwasato T, Itohara S, Li XJ, Mody I, Heintz N, Yang XW (2005). Pathological Cell-Cell Interactions Elicited by a Neuropathogenic Form of Mutant Huntingtin Contribute to Cortical Pathogenesis in HD Mice. Neuron.

[B42] Reddy PH, Williams M, Charles V, Garrett L, Pike-Buchanan L, Whetsell WO, Miller G, Tagle DA (1998). Behavioural abnormalities and selective neuronal loss in HD transgenic mice expressing mutated full-length HD cDNA. Nat Genet.

[B43] Lin CH, Tallaksen-Greene S, Chien WM, Cearley JA, Jackson WS, Crouse AB, Ren S, Li XJ, Albin RL, Detloff PJ (2001). Neurological abnormalities in a knock-in mouse model of Huntington's disease. Hum Mol Genet.

[B44] DiFiglia M, Sapp E, Chase KO, Davies SW, Bates GP, Vonsattel JP, Aronin N (1997). Aggregation of huntingtin in neuronal intranuclear inclusions and dystrophic neurites in brain. Science.

[B45] Gutekunst CA, Li SH, Yi H, Mulroy JS, Kuemmerle S, Jones R, Rye D, Ferrante RJ, Hersch SM, Li XJ (1999). Nuclear and neuropil aggregates in Huntington's disease: relationship to neuropathology. J Neurosci.

[B46] Kuemmerle S, Gutekunst CA, Klein AM, Li XJ, Li SH, Beal MF, Hersch SM, Ferrante RJ (1999). Huntington aggregates may not predict neuronal death in Huntington's disease. Ann Neurol.

[B47] Slow EJ, Graham RK, Osmand AP, Devon RS, Lu G, Deng Y, Pearson J, Vaid K, Bissada N, Wetzel R, Leavitt BR, Hayden MR (2005). Absence of behavioral abnormalities and neurodegeneration in vivo despite widespread neuronal huntingtin inclusions. Proc Natl Acad Sci U S A.

[B48] Saudou F, Finkbeiner S, Devys D, Greenberg ME (1998). Huntingtin acts in the nucleus to induce apoptosis but death does not correlate with the formation of intranuclear inclusions. Cell.

[B49] Arrasate M, Mitra S, Schweitzer ES, Segal MR, Finkbeiner S (2004). Inclusion body formation reduces levels of mutant huntingtin and the risk of neuronal death. Nature.

[B50] Li H, Li SH, Cheng AL, Mangiarini L, Bates GP, Li XJ (1999). Ultrastructural localization and progressive formation of neuropil aggregates in Huntington's disease transgenic mice. Hum Mol Genet.

[B51] Li H, Li SH, Johnston H, Shelbourne PF, Li XJ (2000). Amino-terminal fragments of mutant huntingtin show selective accumulation in striatal neurons and synaptic toxicity. Nat Genet.

[B52] Li H, Li SH, Yu ZX, Shelbourne P, Li XJ (2001). Huntingtin aggregate-associated axonal degeneration is an early pathological event in Huntington's disease mice. J Neurosci.

[B53] Menalled LB, Sison JD, Dragatsis I, Zeitlin S, Chesselet MF (2003). Time course of early motor and neuropathological anomalies in a knock-in mouse model of Huntington's disease with 140 CAG repeats. J Comp Neurol.

[B54] Tallaksen-Greene SJ, Crouse AB, Hunter JM, Detloff PJ, Albin RL (2005). Neuronal intranuclear inclusions and neuropil aggregates in HdhCAG(150) knockin mice. Neuroscience.

[B55] Hodgson JG, Agopyan N, Gutekunst CA, Leavitt BR, LePiane F, Singaraja R, Smith DJ, Bissada N, McCutcheon K, Nasir J, Jamot L, Li XJ, Stevens ME, Rosemond E, Roder JC, Phillips AG, Rubin EM, Hersch SM, Hayden MR (1999). A YAC mouse model for Huntington's disease with full-length mutant huntingtin, cytoplasmic toxicity, and selective striatal neurodegeneration. Neuron.

[B56] Graham RK, Deng Y, Slow EJ, Haigh B, Bissada N, Lu G, Pearson J, Shehadeh J, Bertram L, Murphy Z, Warby SC, Doty CN, Roy S, Wellington CL, Leavitt BR, Raymond LA, Nicholson DW, Hayden MR (2006). Cleavage at the caspase-6 site is required for neuronal dysfunction and degeneration due to mutant huntingtin. Cell.

[B57] Shelbourne PF, Killeen N, Hevner RF, Johnston HM, Tecott L, Lewandoski M, Ennis M, Ramirez L, Li Z, Iannicola C, Littman DR, Myers RM (1999). A Huntington's disease CAG expansion at the murine Hdh locus is unstable and associated with behavioural abnormalities in mice. Hum Mol Genet.

[B58] Wheeler VC, White JK, Gutekunst CA, Vrbanac V, Weaver M, Li XJ, Li SH, Yi H, Vonsattel JP, Gusella JF, Hersch S, Auerbach W, Joyner AL, MacDonald ME (2000). Long glutamine tracts cause nuclear localization of a novel form of huntingtin in medium spiny striatal neurons in HdhQ92 and HdhQ111 knock-in mice. Hum Mol Genet.

[B59] Menalled LB, Sison JD, Wu Y, Olivieri M, Li XJ, Li H, Zeitlin S, Chesselet MF (2002). Early motor dysfunction and striosomal distribution of huntingtin microaggregates in Huntington's disease knock-in mice. J Neurosci.

[B60] Zhou H, Cao FL, Wang ZS, Yu ZX, Nguyen HP, Evans J, Li SH, Li XJ (2003). Huntingtin forms toxic N-terminal fragment complexes that are promoted by the age-dependent decrease in proteasome activity. J Cell Biol.

[B61] Kim YJ, Yi Y, Sapp E, Wang Y, Cuiffo B, Kegel KB, Qin ZH, Aronin N, DiFiglia M (2001). Caspase 3-cleaved N-terminal fragments of wild-type and mutant huntingtin are present in normal and Huntington's disease brains, associate with membranes, and undergo calpain-dependent proteolysis. Proc Natl Acad Sci U S A.

[B62] Gafni J, Ellerby LM (2002). Calpain activation in Huntington's disease. J Neurosci.

[B63] Wellington CL, Ellerby LM, Gutekunst CA, Rogers D, Warby S, Graham RK, Loubser O, van Raamsdonk J, Singaraja R, Yang YZ, Gafni J, Bredesen D, Hersch SM, Leavitt BR, Roy S, Nicholson DW, Hayden MR (2002). Caspase cleavage of mutant huntingtin precedes neurodegeneration in Huntington's disease. J Neurosci.

[B64] Lunkes A, Lindenberg KS, Ben-Haiem L, Weber C, Devys D, Landwehrmeyer GB, Mandel JL, Trottier Y (2002). Proteases acting on mutant huntingtin generate cleaved products that differentially build up cytoplasmic and nuclear inclusions. Mol Cell.

[B65] Hoogeveen AT, Willemsen R, Meyer N, de Rooij KE, Roos RA, van Ommen GJ, Galjaard H (1993). Characterization and localization of the Huntington disease gene product. Hum Mol Genet.

[B66] Kegel KB, Meloni AR, Yi Y, Kim YJ, Doyle E, Cuiffo BG, Sapp E, Wang Y, Qin ZH, Chen JD, Nevins JR, Aronin N, DiFiglia M (2002). Huntingtin is present in the nucleus, interacts with the transcriptional corepressor C-terminal binding protein, and represses transcription. J Biol Chem.

[B67] Tanaka Y, Igarashi S, Nakamura M, Gafni J, Torcassi C, Schilling G, Crippen D, Wood JD, Sawa A, Jenkins NA, Copeland NG, Borchelt DR, Ross CA, Ellerby LM (2006). Progressive phenotype and nuclear accumulation of an amino-terminal cleavage fragment in a transgenic mouse model with inducible expression of full-length mutant huntingtin. Neurobiol Dis.

[B68] Schilling G, Savonenko AV, Klevytska A, Morton JL, Tucker SM, Poirier M, Gale A, Chan N, Gonzales V, Slunt HH, Coonfield ML, Jenkins NA, Copeland NG, Ross CA, Borchelt DR (2004). Nuclear-targeting of mutant huntingtin fragments produces Huntington's disease-like phenotypes in transgenic mice. Hum Mol Genet.

[B69] Benn CL, Landles C, Li H, Strand AD, Woodman B, Sathasivam K, Li SH, Ghazi-Noori S, Hockly E, Faruque SM, Cha JH, Sharpe PT, Olson JM, Li XJ, Bates GP (2005). Contribution of nuclear and extranuclear polyQ to neurological phenotypes in mouse models of Huntington's disease. Hum Mol Genet.

[B70] Cornett J, Cao F, Wang CE, Ross CA, Bates GP, Li SH, Li XJ (2005). Polyglutamine expansion of huntingtin impairs its nuclear export. Nat Genet.

[B71] Sun Y, Savanenin A, Reddy PH, Liu YF (2001). Polyglutamine-expanded huntingtin promotes sensitization of N-methyl-D-aspartate receptors via post-synaptic density 95. J Biol Chem.

[B72] Hackam AS, Singaraja R, Wellington CL, Metzler M, McCutcheon K, Zhang T, Kalchman M, Hayden MR (1998). The influence of huntingtin protein size on nuclear localization and cellular toxicity. J Cell Biol.

[B73] Sugars KL, Rubinsztein DC (2003). Transcriptional abnormalities in Huntington disease. Trends Genet.

[B74] Mantamadiotis T, Lemberger T, Bleckmann SC, Kern H, Kretz O, Martin Villalba A, Tronche F, Kellendonk C, Gau D, Kapfhammer J, Otto C, Schmid W, Schutz G (2002). Disruption of CREB function in brain leads to neurodegeneration. Nat Genet.

[B75] Myers SJ, Dingledine R, Borges K (1999). Genetic regulation of glutamate receptor ion channels. Annu Rev Pharmacol Toxicol.

[B76] Luthi-Carter R, Hanson SA, Strand AD, Bergstrom DA, Chun W, Peters NL, Woods AM, Chan EY, Kooperberg C, Krainc D, Young AB, Tapscott SJ, Olson JM (2002). Dysregulation of gene expression in the R6/2 model of polyglutamine disease: parallel changes in muscle and brain. Hum Mol Genet.

[B77] McCampbell A, Taylor JP, Taye AA, Robitschek J, Li M, Walcott J, Merry D, Chai Y, Paulson H, Sobue G, Fischbeck KH (2000). CREB-binding protein sequestration by expanded polyglutamine. Hum Mol Genet.

[B78] Chai Y, Wu L, Griffin JD, Paulson HL (2001). The role of protein composition in specifying nuclear inclusion formation in polyglutamine disease. J Biol Chem.

[B79] Nucifora FC, Sasaki M, Peters MF, Huang H, Cooper JK, Yamada M, Takahashi H, Tsuji S, Troncoso J, Dawson VL, Dawson TM, Ross CA (2001). Interference by huntingtin and atrophin-1 with cbp-mediated transcription leading to cellular toxicity. Science.

[B80] Steffan JS, Bodai L, Pallos J, Poelman M, McCampbell A, Apostol BL, Kazantsev A, Schmidt E, Zhu YZ, Greenwald M, Kurokawa R, Housman DE, Jackson GR, Marsh JL, Thompson LM (2001). Histone deacetylase inhibitors arrest polyglutamine-dependent neurodegeneration in Drosophila. Nature.

[B81] Huang CC, Faber PW, Persichetti F, Mittal V, Vonsattel JP, MacDonald ME, Gusella JF (1998). Amyloid formation by mutant huntingtin: threshold, progressivity and recruitment of normal polyglutamine proteins. Somat Cell Mol Genet.

[B82] Perez MK, Paulson HL, Pendse SJ, Saionz SJ, Bonini NM, Pittman RN (1998). Recruitment and the role of nuclear localization in polyglutamine-mediated aggregation. J Cell Biol.

[B83] Shimohata T, Nakajima T, Yamada M, Uchida C, Onodera O, Naruse S, Kimura T, Koide R, Nozaki K, Sano Y, Ishiguro H, Sakoe K, Ooshima T, Sato A, Ikeuchi T, Oyake M, Sato T, Aoyagi Y, Hozumi I, Nagatsu T, Takiyama Y, Nishizawa M, Goto J, Kanazawa I, Davidson I, Tanese N (2000). Expanded polyglutamine stretches interact with TAFII130, interfering with CREB-dependent transcription. Nat Genet.

[B84] Hockly E, Richon VM, Woodman B, Smith DL, Zhou X, Rosa E, Sathasivam K, Ghazi-Noori S, Mahal A, Lowden PA, Steffan JS, Marsh JL, Thompson LM, Lewis CM, Marks PA, Bates GP (2003). Suberoylanilide hydroxamic acid, a histone deacetylase inhibitor, ameliorates motor deficits in a mouse model of Huntington's disease. Proc Natl Acad Sci U S A.

[B85] Yu ZX, Li SH, Nguyen HP, Li XJ (2002). Huntingtin inclusions do not deplete polyglutamine-containing transcription factors in HD mice. Hum Mol Genet.

[B86] Kita H, Carmichael J, Swartz J, Muro S, Wyttenbach A, Matsubara K, Rubinsztein DC, Kato K (2002). Modulation of polyglutamine-induced cell death by genes identified by expression profiling. Hum Mol Genet.

[B87] Sipione S, Rigamonti D, Valenza M, Zuccato C, Conti L, Pritchard J, Kooperberg C, Olson JM, Cattaneo E (2002). Early transcriptional profiles in huntingtin-inducible striatal cells by microarray analyses. Hum Mol Genet.

[B88] Dunah AW, Jeong H, Griffin A, Kim YM, Standaert DG, Hersch SM, Mouradian MM, Young AB, Tanese N, Krainc D (2002). Sp1 and TAFII130 transcriptional activity disrupted in early Huntington's disease. Science.

[B89] Li SH, Cheng AL, Zhou H, Lam S, Rao M, Li H, Li XJ (2002). Interaction of Huntington disease protein with transcriptional activator Sp1. Mol Cell Biol.

[B90] Schaffar G, Breuer P, Boteva R, Behrends C, Tzvetkov N, Strippel N, Sakahira H, Siegers K, Hayer-Hartl M, Hartl FU (2004). Cellular toxicity of polyglutamine expansion proteins: mechanism of transcription factor deactivation. Mol Cell.

[B91] Bae BI, Xu H, Igarashi S, Fujimuro M, Agrawal N, Taya Y, Hayward SD, Moran TH, Montell C, Ross CA, Snyder SH, Sawa A (2005). p53 mediates cellular dysfunction and behavioral abnormalities in Huntington's disease. Neuron.

[B92] Lin J, Wu PH, Tarr PT, Lindenberg KS, St-Pierre J, Zhang CY, Mootha VK, Jager S, Vianna CR, Reznick RM, Cui L, Manieri M, Donovan MX, Wu Z, Cooper MP, Fan MC, Rohas LM, Zavacki AM, Cinti S, Shulman GI, Lowell BB, Krainc D, Spiegelman BM (2004). Defects in adaptive energy metabolism with CNS-linked hyperactivity in PGC-1alpha null mice. Cell.

[B93] St-Pierre J, Drori S, Uldry M, Silvaggi JM, Rhee J, Jager S, Handschin C, Zheng K, Lin J, Yang W, Simon DK, Bachoo R, Spiegelman BM (2006). Suppression of reactive oxygen species and neurodegeneration by the PGC-1 transcriptional coactivators. Cell.

[B94] Cui L, Jeong H, Borovecki F, Parkhurst CN, Tanese N, Krainc D (2006). Transcriptional repression of PGC-1alpha by mutant huntingtin leads to mitochondrial dysfunction and neurodegeneration. Cell.

[B95] Weydt P, Pineda VV, Torrence AE, Libby RT, Satterfield TF, Lazarowski ER, Gilbert ML, Morton GJ, Bammler TK, Strand AD, Cui L, Beyer RP, Easley CN, Smith AC, Krainc D, Luquet S, Sweet IR, Schwartz MW, La Spada AR (2006). Thermoregulatory and metabolic defects in Huntington's disease transgenic mice implicate PGC-1alpha in Huntington's disease neurodegeneration. Cell Metab.

[B96] Sanchez I, Xu CJ, Juo P, Kakizaka A, Blenis J, Yuan J (1999). Caspase-8 is required for cell death induced by expanded polyglutamine repeats [see comments]. Neuron.

[B97] Ona VO, Li M, Vonsattel JP, Andrews LJ, Khan SQ, Chung WM, Frey AS, Menon AS, Li XJ, Stieg PE, Yuan J, Penney JB, Young AB, Cha JH, Friedlander RM (1999). Inhibition of caspase-1 slows disease progression in a mouse model of Huntington's disease. Nature.

[B98] Chen M, Ona VO, Li M, Ferrante RJ, Fink KB, Zhu S, Bian J, Guo L, Farrell LA, Hersch SM, Hobbs W, Vonsattel JP, Cha JH, Friedlander RM (2000). Minocycline inhibits caspase-1 and caspase-3 expression and delays mortality in a transgenic mouse model of Huntington disease. Nat Med.

[B99] Li SH, Lam S, Cheng AL, Li XJ (2000). Intranuclear huntingtin increases the expression of caspase-1 and induces apoptosis. Hum Mol Genet.

[B100] Cepeda C, Ariano MA, Calvert CR, Flores-Hernandez J, Chandler SH, Leavitt BR, Hayden MR, Levine MS (2001). NMDA receptor function in mouse models of Huntington disease. J Neurosci Res.

[B101] Song C, Perides G, Liu YF (2002). Expression of full-length polyglutamine-expanded Huntingtin disrupts growth factor receptor signaling in rat pheochromocytoma (PC12) cells. J Biol Chem.

[B102] Zeron MM, Hansson O, Chen N, Wellington CL, Leavitt BR, Brundin P, Hayden MR, Raymond LA (2002). Increased sensitivity to N-methyl-D-aspartate receptor-mediated excitotoxicity in a mouse model of Huntington's disease. Neuron.

[B103] Tang TS, Tu H, Chan EY, Maximov A, Wang Z, Wellington CL, Hayden MR, Bezprozvanny I (2003). Huntingtin and huntingtin-associated protein 1 influence neuronal calcium signaling mediated by inositol-(1,4,5) triphosphate receptor type 1. Neuron.

[B104] Varani K, Rigamonti D, Sipione S, Camurri A, Borea PA, Cattabeni F, Abbracchio MP, Cattaneo E (2001). Aberrant amplification of A(2A) receptor signaling in striatal cells expressing mutant huntingtin. Faseb J.

[B105] Borrell-Pages M, Zala D, Humbert S, Saudou F (2006). Huntington's disease: from huntingtin function and dysfunction to therapeutic strategies. Cell Mol Life Sci.

[B106] Li XJ, Li SH, Sharp AH, Nucifora FC, Schilling G, Lanahan A, Worley P, Snyder SH, Ross CA (1995). A huntingtin-associated protein enriched in brain with implications for pathology. Nature.

[B107] Gauthier LR, Charrin BC, Borrell-Pages M, Dompierre JP, Rangone H, Cordelieres FP, De Mey J, MacDonald ME, Lessmann V, Humbert S, Saudou F (2004). Huntingtin controls neurotrophic support and survival of neurons by enhancing BDNF vesicular transport along microtubules. Cell.

[B108] Engelender S, Sharp AH, Colomer V, Tokito MK, Lanahan A, Worley P, Holzbaur EL, Ross CA (1997). Huntingtin-associated protein 1 (HAP1) interacts with the p150Glued subunit of dynactin. Hum Mol Genet.

[B109] Li SH, Gutekunst CA, Hersch SM, Li XJ (1998). Interaction of huntingtin-associated protein with dynactin P150Glued. J Neurosci.

[B110] McGuire JR, Rong J, Li SH, Li XJ (2006). Interaction of Huntingtin-associated protein-1 with kinesin light chain: implications in intracellular trafficking in neurons. J Biol Chem.

[B111] Li Y, Chin LS, Levey AI, Li L (2002). Huntingtin-associated Protein 1 Interacts with Hepatocyte Growth Factor-regulated Tyrosine Kinase Substrate and Functions in Endosomal Trafficking. J Biol Chem.

[B112] Kittler JT, Thomas P, Tretter V, Bogdanov YD, Haucke V, Smart TG, Moss SJ (2004). Huntingtin-associated protein 1 regulates inhibitory synaptic transmission by modulating gamma-aminobutyric acid type A receptor membrane trafficking. Proc Natl Acad Sci U S A.

[B113] Rong J, McGuire JR, Fang ZH, Sheng G, Shin JY, Li SH, Li XJ (2006). Regulation of intracellular trafficking of huntingtin-associated protein-1 is critical for TrkA protein levels and neurite outgrowth. J Neurosci.

[B114] Chan EY, Nasir J, Gutekunst CA, Coleman S, Maclean A, Maas A, Metzler M, Gertsenstein M, Ross CA, Nagy A, Hayden MR (2002). Targeted disruption of Huntingtin-associated protein-1 (Hap1) results in postnatal death due to depressed feeding behavior. Hum Mol Genet.

[B115] Li SH, Yu ZX, Li CL, Nguyen HP, Zhou YX, Deng C, Li XJ (2003). Lack of huntingtin-associated protein-1 causes neuronal death resembling hypothalamic degeneration in Huntington's disease. J Neurosci.

[B116] Sheng G, Chang GQ, Lin JY, Yu ZX, Fang ZH, Rong J, Lipton SA, Li SH, Tong G, Leibowitz SF, Li XJ (2006). Hypothalamic huntingtin-associated protein 1 as a mediator of feeding behavior. Nat Med.

[B117] Petersen A, Gil J, Maat-Schieman ML, Bjorkqvist M, Tanila H, Araujo IM, Smith R, Popovic N, Wierup N, Norlen P, Li JY, Roos RA, Sundler F, Mulder H, Brundin P (2005). Orexin loss in Huntington's disease. Hum Mol Genet.

[B118] Kalchman MA, Koide HB, McCutcheon K, Graham RK, Nichol K, Nishiyama K, Kazemi-Esfarjani P, Lynn FC, Wellington C, Metzler M, Goldberg YP, Kanazawa I, Gietz RD, Hayden MR (1997). HIP1, a human homologue of S. cerevisiae Sla2p, interacts with membrane-associated huntingtin in the brain. Nat Genet.

[B119] Metzler M, Li B, Gan L, Georgiou J, Gutekunst CA, Wang Y, Torre E, Devon RS, Oh R, Legendre-Guillemin V, Rich M, Alvarez C, Gertsenstein M, McPherson PS, Nagy A, Wang YT, Roder JC, Raymond LA, Hayden MR (2003). Disruption of the endocytic protein HIP1 results in neurological deficits and decreased AMPA receptor trafficking. Embo J.

[B120] Mishra SK, Agostinelli NR, Brett TJ, Mizukami I, Ross TS, Traub LM (2001). Clathrin- and AP-2-binding sites in HIP1 uncover a general assembly role for endocytic accessory proteins. J Biol Chem.

[B121] Waelter S, Scherzinger E, Hasenbank R, Nordhoff E, Lurz R, Goehler H, Gauss C, Sathasivam K, Bates GP, Lehrach H, Wanker EE (2001). The huntingtin interacting protein HIP1 is a clathrin and alpha-adaptin-binding protein involved in receptor-mediated endocytosis. Hum Mol Genet.

[B122] Gunawardena S, Her LS, Brusch RG, Laymon RA, Niesman IR, Gordesky-Gold B, Sintasath L, Bonini NM, Goldstein LS (2003). Disruption of axonal transport by loss of huntingtin or expression of pathogenic polyQ proteins in Drosophila. Neuron.

[B123] Trushina E, Dyer RB, Badger JD, Ure D, Eide L, Tran DD, Vrieze BT, Legendre-Guillemin V, McPherson PS, Mandavilli BS, Van Houten B, Zeitlin S, McNiven M, Aebersold R, Hayden M, Parisi JE, Seeberg E, Dragatsis I, Doyle K, Bender A, Chacko C, McMurray CT (2004). Mutant huntingtin impairs axonal trafficking in Mammalian neurons in vivo and in vitro. Mol Cell Biol.

[B124] Parker JA, Connolly JB, Wellington C, Hayden M, Dausset J, Neri C (2001). Expanded polyglutamines in Caenorhabditis elegans cause axonal abnormalities and severe dysfunction of PLM mechanosensory neurons without cell death. Proc Natl Acad Sci U S A.

[B125] Szebenyi G, Morfini GA, Babcock A, Gould M, Selkoe K, Stenoien DL, Young M, Faber PW, MacDonald ME, McPhaul MJ, Brady ST (2003). Neuropathogenic forms of huntingtin and androgen receptor inhibit fast axonal transport. Neuron.

[B126] Li H, Wyman T, Yu ZX, Li SH, Li XJ (2003). Abnormal association of mutant huntingtin with synaptic vesicles inhibits glutamate release. Hum Mol Genet.

[B127] Li XJ, Li SH (2005). HAP1 and intracellular trafficking. Trends Pharmacol Sci.

[B128] Chang DT, Rintoul GL, Pandipati S, Reynolds IJ (2006). Mutant huntingtin aggregates impair mitochondrial movement and trafficking in cortical neurons. Neurobiol Dis.

[B129] Lee WC, Yoshihara M, Littleton JT (2004). Cytoplasmic aggregates trap polyglutamine-containing proteins and block axonal transport in a Drosophila model of Huntington's disease. Proc Natl Acad Sci U S A.

[B130] Coleman MP, Perry VH (2002). Axon pathology in neurological disease: a neglected therapeutic target. Trends Neurosci.

[B131] Raff MC, Whitmore AV, Finn JT (2002). Axonal self-destruction and neurodegeneration. Science.

[B132] Rothstein JD, Dykes-Hoberg M, Pardo CA, Bristol LA, Jin L, Kuncl RW, Kanai Y, Hediger MA, Wang Y, Schielke JP, Welty DF (1996). Knockout of glutamate transporters reveals a major role for astroglial transport in excitotoxicity and clearance of glutamate. Neuron.

[B133] Ridet JL, Malhotra SK, Privat A, Gage FH (1997). Reactive astrocytes: cellular and molecular cues to biological function. Trends Neurosci.

[B134] Fields RD, Stevens-Graham B (2002). New insights into neuron-glia communication. Science.

[B135] Beal MF (1994). Huntington's disease, energy, and excitotoxicity. Neurobiol Aging.

[B136] Coyle JT, Schwarcz R (1976). Lesion of striatal neurones with kainic acid provides a model for Huntington's chorea. Nature.

[B137] DiFiglia M (1990). Excitotoxic injury of the neostriatum: a model for Huntington's disease. Trends Neurosci.

[B138] Choi DW (1988). Glutamate neurotoxicity and diseases of the nervous system. Neuron.

[B139] Hantraye P, Riche D, Maziere M, Isacson O (1990). A primate model of Huntington's disease: behavioral and anatomical studies of unilateral excitotoxic lesions of the caudate-putamen in the baboon. Exp Neurol.

[B140] Greene JG, Porter RH, Eller RV, Greenamyre JT (1993). Inhibition of succinate dehydrogenase by malonic acid produces an "excitotoxic" lesion in rat striatum. J Neurochem.

[B141] Levine MS, Klapstein GJ, Koppel A, Gruen E, Cepeda C, Vargas ME, Jokel ES, Carpenter EM, Zanjani H, Hurst RS, Efstratiadis A, Zeitlin S, Chesselet MF (1999). Enhanced sensitivity to N-methyl-D-aspartate receptor activation in transgenic and knockin mouse models of Huntington's disease. J Neurosci Res.

[B142] Laforet GA, Sapp E, Chase K, McIntyre C, Boyce FM, Campbell M, Cadigan BA, Warzecki L, Tagle DA, Reddy PH, Cepeda C, Calvert CR, Jokel ES, Klapstein GJ, Ariano MA, Levine MS, DiFiglia M, Aronin N (2001). Changes in cortical and striatal neurons predict behavioral and electrophysiological abnormalities in a transgenic murine model of Huntington's disease. J Neurosci.

[B143] Maragakis NJ, Rothstein JD (2001). Glutamate transporters in neurologic disease. Arch Neurol.

[B144] Lievens JC, Woodman B, Mahal A, Spasic-Boscovic O, Samuel D, Kerkerian-Le Goff L, Bates GP (2001). Impaired glutamate uptake in the R6 Huntington's disease transgenic mice. Neurobiol Dis.

[B145] Behrens PF, Franz P, Woodman B, Lindenberg KS, Landwehrmeyer GB (2002). Impaired glutamate transport and glutamate-glutamine cycling: downstream effects of the Huntington mutation. Brain.

[B146] Lievens JC, Rival T, Iche M, Chneiweiss H, Birman S (2005). Expanded polyglutamine peptides disrupt EGF receptor signaling and glutamate transporter expression in Drosophila. Hum Mol Genet.

[B147] Shin JY, Fang ZH, Yu ZX, Wang CE, Li SH, Li XJ (2005). Expression of mutant huntingtin in glial cells contributes to neuronal excitotoxicity. J Cell Biol.

